# PIM1 destabilization activates a p53-dependent response to ribosomal stress in cancer cells

**DOI:** 10.18632/oncotarget.8070

**Published:** 2016-03-14

**Authors:** Vinay Sagar, Sara Caldarola, Valentina Aria, Valentina Monteleone, Claudia Fuoco, Cesare Gargioli, Stefano Cannata, Fabrizio Loreni

**Affiliations:** ^1^ Department of Biology, University of Rome Tor Vergata, Roma, Italy

**Keywords:** PIM1, MDM2, AKT, p53, ribosomal stress

## Abstract

Defects in ribosome biogenesis triggers a stress response (ribosomal stress) that can lead to growth arrest and apoptosis. Signaling pathways activated by ribosomal stress are specifically involved in the pathological mechanism of a group of disorders defined as ribosomopathies. However, more generally, the quality control of ribosome synthesis is part of the regulatory circuits that control cell metabolism. A number of studies identified tumor suppressor p53 as a central player in ribosomal stress. We have previously reported that the kinase PIM1 plays a role as a sensor for ribosome deficiency. In this report we address the relationship between PIM1 and p53 in cancer cell lines after depletion of a ribosomal protein. We identified a novel signaling pathway that includes the kinase AKT and the ubiquitin ligase MDM2. In fact, our results indicate that the lower level of PIM1, induced by ribosomal stress, causes inactivation of AKT, inhibition of MDM2 and a consequent p53 stabilization. Therefore, we propose that activation of p53 in response to ribosomal stress, is dependent on the pathway PIM1-AKT-MDM2. In addition, we report evidence that PIM1 level may be relevant to assess the sensitivity of cancer cells to chemotherapeutic drugs that induce ribosomal stress.

## INTRODUCTION

Ribosome biogenesis is a fundamental cellular process that requires a substantial energetic investment. It involves the activity of the three RNA polymerases and nearly 200 non-ribosomal factors necessary for the synthesis, maturation and export of the subunits [1]. Multiple regulatory mechanisms are likely to control the synthesis and the quality of final products and a defect at any step of the process activates a cellular response defined as ribosomal stress. Malfunction of ribosome biogenesis is at the basis of a group of disorders named ribosomopathies, characterized by a broad range of tissue-specific clinical phenotypes [2–4]. In addition, altered synthesis and function of the translation apparatus have been linked to predisposition to cancer development [5–7].

Ribosomal stress has been shown to trigger signaling pathways that, by activating the tumor suppressor p53, lead to cell cycle arrest and apoptosis [8, 9]. However p53-independent mechanisms have also been described [10–12]. A considerable number of studies indicate that defects in ribosome production, such as ribosomal protein (RP) insufficiency, rRNA transcription inhibition and block of ribosome subunits export, lead to p53 upregulation [13–20]. The central role of p53 has been confirmed in animal models where the phenotypic effects of ribosomal stress could be attenuated in a p53-null genetic background [16, 17, 21, 22].

One of the key regulator of p53 is the E3 ubiquitin ligase MDM2 [23]. In growing cells MDM2 binds p53, inhibiting its transactivation function and targeting it to proteasome degradation both in the nucleus and cytoplasm [24]. p53 activity is also regulated by MDM4 (also called MDMX), a MDM2 homolog [25]. MDM4 forms hetero-oligomers with MDM2 and can stimulate p53 ubiquitination and degradation [26]. Oncogene activation, DNA damage, hypoxia etc., inhibit MDM2-p53 interaction. Consequently, p53 is rapidly stabilized leading to a block of proliferation and apoptosis. It has been proposed that activation of p53 in response to ribosomal stress depends on the interaction between some RPs and MDM2 [8, 9]. According to this model, upon inhibition of ribosome biogenesis, RPs bind MDM2 in the nucleoplasm blocking its ubiquitin ligase activity and promoting p53 stabilization. The list of RPs able to bind MDM2 is still growing but RPL5 and RPL11 seem to have a more important role [18, 27–29]. Different hypotheses have been proposed on how the RP-MDM2 interaction occurs in the cell. A few studies suggested that nucleolar disruption allows the passive diffusion of free RPs from the nucleolus into the nucleoplasm where they would bind MDM2 [19, 30, 31]. In addition, it has been proposed that it is the complex RPL5/RPL11/5S rRNA that interacts with MDM2 [32, 33]. It has also been showed that impaired 40S biogenesis induces a selective translational upregulation of RP mRNAs characterized by a Terminal Oligopyrimidine (TOP) stretch at the 5′ end [18, 34]. Overproduced RPL11 would thus enter the nucleus and bind to MDM2 stabilizing p53 [35].

Other proteins have been proposed to have a role in the response to ribosomal stress. In particular, we reported a role of the oncogene PIM1 in regulating cell cycle and proliferation in hematopoietic cells with induced RPS19 deficiency [36]. PIM1 (proviral insertion site in Moloney Murine Leukemia Virus) is a serine-threonine kinase regulated by a variety of growth factors and cytokines that belongs to the family of Pim kinases (together with Pim2 and Pim3). It is highly expressed in hematopoietic and epithelial cells as well as in prostate cancer and other tumors [37–39]. PIM1 has been reported to target a number of substrates involved in the regulation of the cell cycle, apoptosis and energy metabolism [40–42]. Moreover, there is evidence of a functional interaction between PIM1 and AKT, which is key component of the PI3K/mTOR signaling pathway and a major regulator of protein synthesis [43, 44]. Interestingly, AKT also modulates the stability of p53 by phosphorylating and activating MDM2 [45, 46].

We have previously shown that PIM1 interacts with RPS19 [47] and cosediments with ribosomal particles in sucrose gradients of hematopoietic cell extracts. Interaction with the ribosome may increase the stability of PIM1 whereas defective ribosome synthesis induces a destabilization of this kinase, with a consequent increase in the level of the cell cycle inhibitor p27Kip1 and a block of cell proliferation. Importantly, this occurs even in a p53-negative background [36].

We have now found that, in cancer cells of different origins, PIM1 destabilization in response to ribosomal stress, can generate p53 activation through a pathway that involves AKT and MDM2. According to these findings, we observed that a higher level of PIM1 can protect cancer cells from the inhibitory effects of chemotherapeutic drugs which induce ribosomal stress, such as doxorubicin and cisplatin.

## RESULTS

### PIM1 cosediments with ribosomes

**Figure 1:**
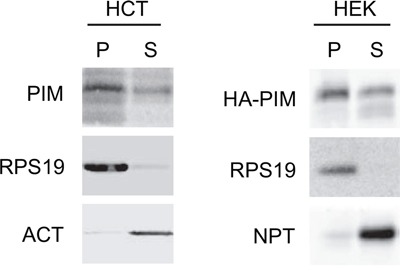
PIM1 association with the ribosome Cytoplasmic extracts from HCT116 and HEK293 transfected with HA-PIM1 were separated by ultra- centrifugation to a pellet (P), containing ribosomes and ribosomal subunits and a supernatant (S), containing free cytoplasmic proteins. The two fractions were analyzed by western blot with primary antibodies against PIM1, RPS19, β-Actin (ACT) and Neomycin phosphotransferase (NPT, encoded by the expression vector). The loading ratio between P and S was 3:1. In the left panel, PIM1 antibody detects endogenous protein whereas in the right panel the transfected HA-PIM1.

We have previously reported evidence that endogenous PIM1 interacts with ribosomes in HEK293 [47], K562 and TF-1 erythroid cells [36]. To verify that this occurs in other cell types and with exogenous protein, we prepared cytoplasmic extracts from HCT116 cells and from HEK293 cells expressing HA-tagged PIM1. Extracts were separated by ultracentrifugation into two fractions: 1) P, pellet, which includes polysomes and ribosomal subunits and 2) S, supernatant, which includes free cytosolic proteins. Western blot analysis of fractionated extracts (Figure 1), showed that an evident part of cytoplasmic PIM1 is observed in the polysomal pellet in both cell lines. This indicates that, similarly to erythroid cells, PIM1 cosediments with ribosomes possibly due to the interaction with RPS19.

### Depletion of a ribosomal protein causes a decrease of PIM1 and an increase of p53 levels

**Figure 2:**
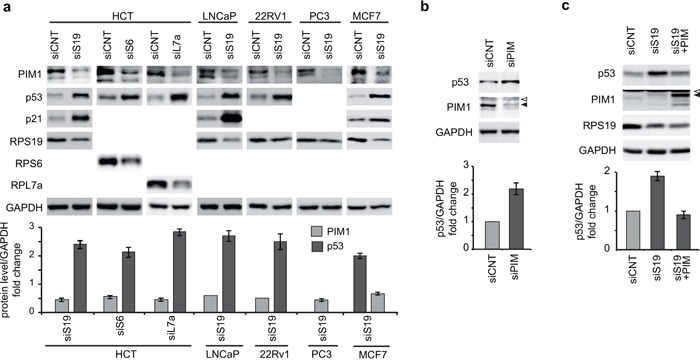
Analysis of PIM1 and p53 levels Total extracts were analyzed by western blot with indicated primary antibodies. Quantification of proteins from at least three independent experiments are reported in lower panels as a column plot of the mean±s.e.m. of the densitometry values normalized by GAPDH. **a.** Extracts from HCT116, LNCaP, 22Rv1, PC3 and MCF7 cells transfected with indicated siRNA (PIM1 analysis in LNCaP and 22Rv1 is the mean of two experiments). **b.** Extracts from HCT116 cells transfected with control (siCNT) or with PIM1-specific siRNA (siPIM). **c.** Extracts from HCT116 cells transfected first with RPS19 specific siRNA (siS19) and then transduced with PIM1-expressing lentivirus. Open triangles indicate non-specific bands; filled triangles indicate PIM1 bands.

We monitored the level of PIM1 and p53 in response to RP depletion, in a number of cell lines of different origins and with different endogenous levels of both proteins. The efficiency of RNAi for RPS19 was preliminarly tested in HCT116 cells and it is reported in Supplementary Figure S1. Then, we analyzed protein levels in the following cell lines: HCT116 (colon cancer), LNCaP, PC3, 22Rv1 (prostate cancer), MCF7 (breast cancer). PC3 cells do not express p53. All the cell lines were transfected with siRNA specific for RPS19, or an unrelated siRNA as a control. In addition, HCT116 cells were transfected with siRNA specific for RPS6 and RPL7a. The results of our analysis, reported in Figure 2a, indicate that depletion of an RP (RPS19 or RPS6 or RPL7a) causes a decrease of PIM1 level and an increase of p53 (if present) in all cell lines tested.

To address if a causal relationship could be established between alteration in the level of PIM1 and p53, we set up the following experiments in HCT116 cells: 1) PIM1 inhibition by siRNA 2) PIM1 overexpression during ribosomal stress. In this second experiment, cells were first transfected with siRNA specific for RPS19 to induce ribosomal stress and, after 24 h, were transduced with a PIM1-expressing lentivirus. In both cases the level of relevant proteins were monitored by western blot with specific antibodies. The results, shown in Figure 2b and 2c, indicate that the reduction of PIM1 level is sufficient to cause an increase in p53 levels. Consistent with this data, treatment of HCT116 cells with PIM inhibitor SMI-4a induces an increase of p53 level (Supplementary Figure S2). Finally, overexpression of PIM1 rescues the effect of RPS19 depletion and restores p53 to control levels (Figure 2c). As a control for this last experiment, we overexpressed PIM1 in control HCT cells. The results (Supplementary Figure S3) show that in this case PIM1 does not affect p53, possibly because its level is already low in control cells.

### AKT phosphorylation decreases during ribosomal stress and is dependent on PIM1 levels

**Figure 3:**
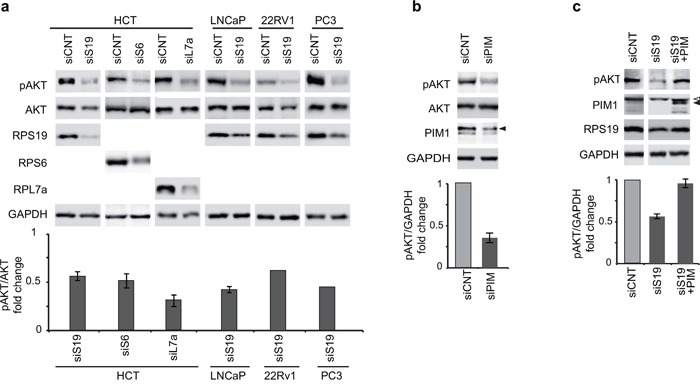
Phosphorylation of AKT at Ser473 Total extracts were analyzed by western blot with indicated primary antibodies. Quantification of the signal was carried out as in Figure 2. **a.** Extracts from HCT116, LNCaP, 22Rv1 and PC3 cells transfected with indicated siRNA (analysis in 22Rv1 and PC3 cells is the mean of two experiments). **b.** Extracts from HCT116 cells transfected with control (siCNT) or with PIM1-specific siRNA (siPIM). **c.** Extracts from HCT116 cells transfected first with control (siCNT) or RPS19 specific siRNA (siS19) and then transduced with PIM1-expressing lentivirus. <, non-specific band. The gap between the lanes indicates that part of the gel (containing additional controls) has been eliminated. Open triangles indicate non-specific bands; filled triangles indicate PIM1 bands.

To identify additional components that could be involved in the functional interaction between PIM1 and p53 in response to ribosomal stress, we addressed AKT. In fact, the activity of this kinase has been shown to be inhibited by RP depletion [48] and AKT has also been indicated as a regulator of p53 [45]. We observed that RPS19 depletion in HCT116, LNCaP, PC3 and 22Rv1 cell lines, as well as RPS6 and RPL7a depletion in HCT116 cells, induce a decrease of AKT phosphorylation on Ser473 (Figure 3a). To investigate the possible role of PIM1 reduction in the dephosphorylation of AKT, we performed the following experiments (similar to those described in the previous paragraph): 1) PIM1 depletion by siRNA transfection in HCT116 cells, 2) PIM1 overexpression during ribosomal stress. This second experiment was performed, similarly to the one described in the previous paragraph, by transfecting the cells with siRNA specific for RPS19 and then, with PIM1-expressing lentivirus. The results, shown in Figure 3b and 3c, indicate that PIM1 depletion causes a decrease of AKT phosphorylation on Ser473. In addition, restoring PIM1 level after RPS19 depletion causes a recovery of Ser473 phospho-AKT to control level. The effect of PIM1 overexpression on the level of Ser473 phospho-AKT can be observed also in control HCT cells (Supplementary Figure S3).

### MDM2 and p53 levels are affected by AKT dephosphorylation

**Figure 4:**
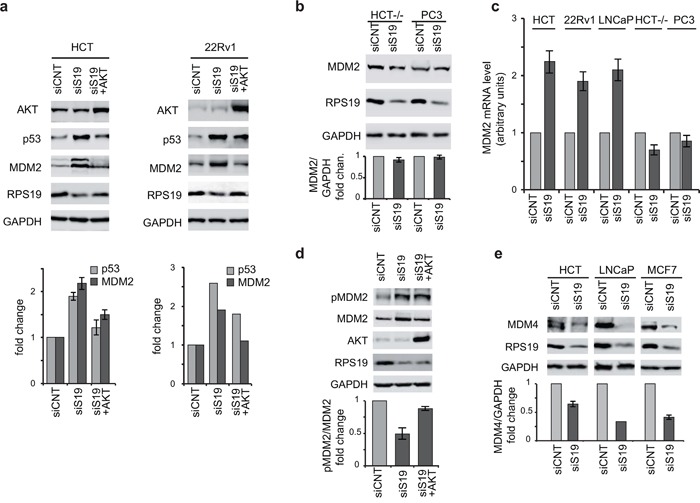
AKT regulate p53 and MDM2 Total protein extracts were analyzed by western blot with indicated primary antibodies. Quantification of the signal was carried out as in Figure 2. **a.** Extracts from HCT116 and 22Rv1 cells transfected with control siRNA (siCNT), with RPS19-specific siRNA (siS19), or with RPS19-specific siRNA plus AKT expressing plasmid (siS19+AKT). Quantification in 22Rv1 cells is from two independent experiment. **b.** Extracts from HCT−/− and PC3 cells transfected with control siRNA (siCNT) or with RPS19-specific siRNA (siS19). **c.** Total RNA was extracted from HCT116, 22Rv1, LNCaP, HCT−/− and PC3 cells transfected with control siRNA (siCNT) or with RPS19-specific siRNA (siS19) and analyzed by qRT-PCR with primers specific for MDM2 and GAPDH. The results of triplicate RT–qPCR from three independent RNA preparations are reported as a column plot of the mean±s.e.m. of MDM2 mRNA normalized by GAPDH mRNA. **d.** Extracts from HCT116 cells transfected with control siRNA (siCNT), with RPS19-specific siRNA (siS19) or with RPS19-specific siRNA plus AKT expressing plasmid (siS19+AKT). **e.** Extracts from HCT116, LNCaP and MCF7 cells transfected with control siRNA (siCNT) or with RPS19-specific siRNA (siS19).

Previous studies have shown that AKT can phosphorylate MDM2 leading to p53 destabilization [45]. Therefore, it could be hypothesized that the increase of p53 in response to ribosomal stress could be dependent on AKT dephosphorylation (described in the previous paragraph). For this reason we addressed the role of AKT in the regulation of p53 and MDM2 in our experimental setup. HCT116 and 22Rv1 cells were firstly transfected with siRNA specific for RPS19 to induce ribosomal stress followed by a plasmid expressing a constitutively active form of AKT (E40K). As shown in Figure 4a, overexpression of active AKT in RPS19-depleted cells rescues p53 to control levels. In the same experiment we also monitored MDM2 level. We observed that RPS19 depletion induces a significant increase in MDM2 whereas AKT overexpression causes a recovery of MDM2 to control level.

Since transcriptional activity of the MDM2 gene is upregulated by p53, we hypothesized that the observed increment of MDM2 in RPS19-depleted cells could occur at the transcriptional level and could be dependent on p53. To verify this model, we induced RPS19 depletion in the following cell lines: HCT116, LNCaP, 22Rv1 (all expressing p53), HCT116 −/−, PC3 (p53-negative cells). We analyzed MDM2 mRNA and protein levels by qRT-PCR and western blot, respectively. The results, shown in Figure 4b and 4c, indicate that RPS19 depletion 1) does not affect MDM2 protein levels in p53-negative cells and 2) causes an increase of MDM2 mRNA level in p53-expressing cells (HCT116, LNCaP, 22Rv1) but not in p53-negative cells (HCT116−/−, PC3).

It has been shown that AKT-dependent phosphorylation on Ser166 promotes MDM2 nuclear translocation [49]. Therefore AKT dephosphorylation observed during ribosomal stress could inhibit MDM2 import in the nucleus and as a consequence cause p53 stabilization. In addition, MDM2 increase could induce MDM4 degradation [24]. For these reasons, to further investigate the paradoxical observation of increased levels of both MDM2 and p53, we addressed the phosphorylation level of MDM2 on Ser166 (target of AKT) and the expression of MDM4 during ribosomal stress.

RPS19 was depleted in HCT116, LNCaP and MCF7 cells. HCT116 cells were then transfected with a plasmid expressing a constitutively active form of AKT (E40K) and the levels of Ser166 phopsho-MDM2 were analyzed by western blot (Figure 4d). All the cell lines were also analyzed for MDM4 level (Figure 4e). As reported in Figure 4d and 4e, RPS19 deficiency causes 1) a decrease of p-MDM2 relative to total MDM2 that is recovered by AKT overexpression and 2) a decrease of MDM4 level. These results are consistent with the following hypothesis: AKT inactivation causes a decrease of p-MDM2 and as a consequence, an increase of p53. Higher p53 level causes an increase of MDM2 which, in turn, induces a degradation of MDM4.

### MDM2 subcellular localization during ribosomal stress

The current model indicates that p53 protein is normally maintained at a low level in the cell due to its interaction with MDM2 which causes its ubiquitination and degradation through the 26S proteasome. Both p53 and MDM2 possess nuclear export signals and shuttling of MDM2 from the nucleus to the cytoplasm appears to be important for p53 ubiquitination [24].

**Figure 5:**
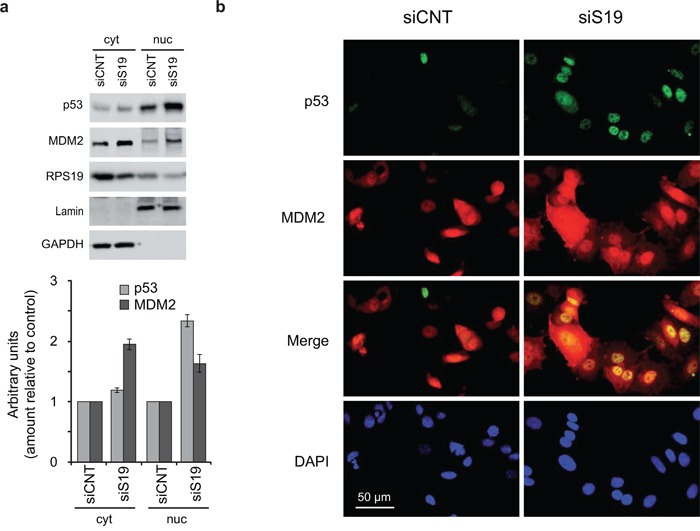
Localization of p53 and MDM2 **a.** Cytoplasmic (cyt) and nuclear (nuc) extracts from HCT116 cells transfected with control (siCNT) or with RPS19-specific siRNA (siS19) were analyzed by western blot with primary antibodies for p53, MDM2, RPS19, Lamin and GAPDH. Quantification from three independent experiments is reported in lower panel as column plot of the mean±s.e.m of the values normalized by control. **b.** MCF7 cells, transfected with control (siCNT) or with RPS19-specific siRNA (siS19), were fixed and stained with antibodies for p53 and MDM2 and then incubated with FITC–conjugated anti-Rabbit IgG and TRITC-conjugated anti-Moiuse IgG,. The localization of p53 and MDM2 was visualized by fluorescence microscopy.

For these reasons we addressed the subcellular localization of both p53 and MDM2 in response to ribosomal stress. HCT116 cells were treated with siRNA specific for RPS19 for 48 h and cell extracts were separated into nuclear and cytoplasmic fractions and analyzed by western blot. The results, reported in Figure 5a, show that in response to RPS19 depletion, p53 level increases more clearly in the nucleus and partly in the cytoplasm, whereas the increase of MDM2 is mostly visible in the cytoplasm.

Subcellular localization of p53 and MDM2 was also analyzed by immunofluorescence. For this purpose, MCF7 cells were treated with control or RPS19-specific siRNA and, after incubation with appropriate antibodies, were analyzed by fluorescence microscopy. The results (Figure 5b) show that, consistent with the biochemical analysis reported in Figure 5a, following RPS19 depletion, the p53 increase occurs mainly in the nucleus whereas MDM2 is predominantly increased in the cytoplasm.

### PIM1 overexpression confers resistance to chemotherapeutic drugs that induce ribosomal stress

A large variety of chemotherapeutic drugs act by inhibition of ribosome biogenesis at different steps [50] therefore eliciting ribosomal stress. Since we propose here that PIM1 mediates the response to ribosomal defects, we decided to verify if modification of PIM1 level could alter the sensitivity of cultured cells to chemotherapeutic drugs.

First, we decided to confirm that the alterations induced by RP depletion could be recapitulated by treatment with an inhibitor known to induce ribosomal stress. For this purpose, HCT116 cells were treated for 4 hrs with 50 nM Actinomycin D and protein level was analyzed by Western blot. The results, reported in Supplementary Figure S4, shows that, similarly to RPS19 depletion, Actinomycin D treatment induces 1) PIM1 decrease, 2) Ser473 phospho-AKT decrease and 3) p53 increase.

**Figure 6:**
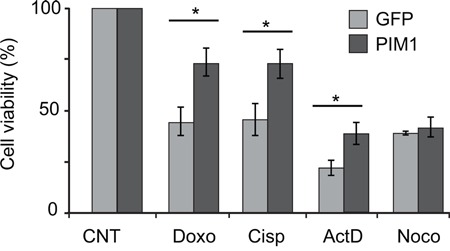
PIM1 overexpression causes reduced sensitivity to chemoterapeutic drugs HCT116 cells transduced with PIM1-expressing lentivirus, were treated with Doxorubicin, Cisplatin, Actinomycin D or Nocodazole. After 48 h treatment, cell viability was assessed by MTT assays. Quantification of cell viability from three independent experiments is reported as a column plot of the mean ±s.e.m. of the values normalized by control. *, P<0.05.

HCT116 cells were transduced with PIM1-expressing lentivirus or with a GFP-expressing lentivirus as a control. Cells were then treated for 48 h with Cisplatin, Doxorubicin and Actinomycin D, both of which are known to affect ribosomal RNA synthesis at different steps. In addition, cells were also treated with Nocodazole, a cell cycle inhibitor which does not affect ribosome biogenesis. Consistent with our hypothesis, the analysis of cell viability by MTT assay, reported in Figure 6, shows that PIM1 overexpression renders the cells less sensitive to the inhibitory effects of Cisplatin, Doxorubicin and Actinomycin D, but does not change the response to Nocodazole.

## DISCUSSION

We report here the identification of a novel signaling pathway activated by ribosome synthesis defects. It involves the two oncogenic kinases PIM1 and AKT and the tumor suppressor p53. The role of this pathway is to activate a growth inhibitory response to align cellular metabolism with the functionality of the translational apparatus.

PIM1 has been identified as an important player in the control of cell growth and survival in hematopoietic, colon and prostate cancers [51]. Previous findings from our group showed that ribosomal stress, induced by depletion of RPS19, causes cell cycle arrest and a block in cell proliferation due to destabilization of PIM1 [36]. On the other hand, a number of studies reported the activation of p53 in response to defects in ribosome synthesis (see introduction). For these reasons, we decided to address the relationship between PIM1 and p53 in the response to depletion of RPs in cultured cells. To avoid possible cell type specific effects and to focus on general mechanisms, we used a number of cell lines of different origins: colon (HCT116), prostate (22Rv1, LNCaP, PC3), and mammary gland (MCF7). After confirming the previously reported interaction of PIM1 with the ribosome, we analyzed the level of both PIM1 and p53 during conditions of RP deficiency (RPS19 or RPS6 or RPL7a). The fact that depletion of different RPs elicits a similar response suggests a common regulatory mechanism. Moreover, in all the experiments, we observed an inverse correlation between the levels of the two proteins: PIM1 decreased to about 50% whereas p53 increased 2-3 fold compared to control. Importantly, we could establish a causal relationship between the two observations on the basis of the following results: 1) depletion of PIM1 by siRNA in HCT116 cells induced an increase of p53 and 2) restoring the level of PIM1 in RPS19-depleted cells caused a consequent reduction of p53 to control levels (Figure 2).

Subsequently, we analyzed the possible role of AKT as an intermediate between PIM1 and p53. Our results were consistent with the hypothesis that, in response to ribosomal stress, PIM1 levels affect the phosphorylation of AKT on Ser473. In fact, RP depletion induces a coordinated decrease of PIM1 and phospho-AKT levels in all the cell lines analyzed. Moreover, we observed that 1) PIM1 depletion causes a decrease of phospho-AKT and 2) overexpression of PIM1 during ribosomal stress induces a recovery of phospho-AKT (Figure 3c). To understand how AKT dephosphorylation could affect p53, we included MDM2 and MDM4 in our analysis. In fact, a complex regulatory interaction exists among p53, MDM2 and MDM4 [24]. The main features of this network, relevant for our analysis, are: 1) MDM2 is an ubiquitin E3 ligase that promotes p53 degradation but it is also a transcriptional target of p53, resulting in a feedback loop; 2) activated AKT can phosphorylate MDM2 on Ser166 and Ser186, stimulating nuclear localization and ubiquitylating activity; 3) MDM4 forms hetero-oligomers with MDM2 that enhance ubiquitylation and degradation of p53; 4) MDM4 is a direct substrate of MDM2 for targeted ubiquitylation and degradation.

**Figure 7:**
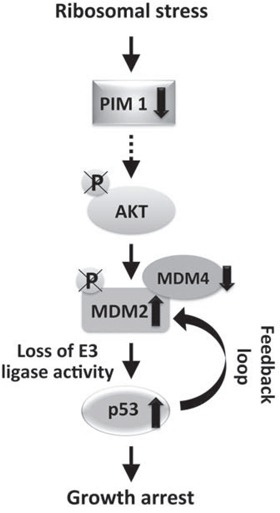
Schematic of the signaling pathway activated by ribosomal stress Ribosomal stress, due to RP depletion, induces a decrease of PIM1 level that inhibits phosphorylation of AKT on Ser473; the consequent AKT inactivation causes a dephosphorylation of MDM2, inhibiting its nuclear import and ubiquitin ligase activity. This generates a stabilization of p53 that, through the feedback loop, induces an increase of total MDM2 level. The high level of MDM2 causes degradation of MDM4 which produces a further stabilization of p53. The final outcome of this signaling pathway is a p53-dependent cell growth inhibition.

Our analysis showed that RPS19 depletion causes an increase of total MDM2 level but a decrease of the phosphorylated (Ser166) fraction. The increase of MDM2 is dependent on p53 as it does not occur in p53-negative cells. In addition, the level of MDM4 is reduced to about 50% with respect to control. These results are consistent with the following model, as shown in Figure 7: ribosomal stress, due to RP depletion, induces a decrease of PIM1 levels that inhibits phosphorylation of AKT on Ser473; the consequent AKT inactivation causes a dephosphorylation of MDM2, inhibiting its nuclear import and ubiquitin ligase activity. This results in stabilization of p53 that, through the feedback loop, induces an increase of total MDM2 levels. The high level of MDM2 causes degradation of MDM4 leading to a further stabilization of p53. The final outcome of this signaling pathway is p53-dependent cell growth inhibition.

Finally, we found evidence that the pathway we have presented here, may be involved in the cell response to a number of chemotherapeutic drugs. In fact, overexpression of PIM1 renders cultured cells less sensitive to drugs which inhibit ribosome synthesis. Thus the data suggests that the measurement of the cellular PIM1 level is critical for the selection of the most appropriate chemotherapeutic agent during cancer treatment.

We are aware that our hypothesized mechanism needs further clarification. For instance, it is not clear how the decrease of PIM1 causes AKT dephosphorylation on Ser473 and if the interaction of PIM1 with the ribosome plays a role. An effect of PIM1 on AKT phosphorylation has been reported in at least two other independent studies, [44, 52] but the mechanism has not been addressed. One possibility is the involvement of mTORC2, which is already known to interact with the ribosome [48]. In fact, Zinzalla and colleagues showed that depletion of an RP (RPL7 or RPS16) reduced mTORC2 kinase activity and mTORC2 signaling, possibly due to decreased interaction with the ribosome. We hypothesize that PIM1 destabilization during ribosomal stress plays a role in this inhibition, but further studies are needed to verify this mechanism.

Another question that remains to be clarified is the relationship between the pathway we propose and the model involving the interaction of RPs with MDM2 described in a number of publications (see Introduction). We think that the two mechanisms could both participate in the response to ribosomal stress. The pathway PIM1-AKT-MDM2, described here, is active in the cytoplasm, whereas the interaction RPs-MDM2 occurs in the nucleus. The prevalence of one or the other could depend on different circumstances (kind of stress, cell type etc.).

## MATERIALS AND METHODS

### Cell culture and transfection

Human colorectal carcinoma HCT116 (HCT), Human prostate cancer 22Rv1 and PC3, Human breast adenocarcinoma MCF7 and Human embryonic kidney HEK293, cells were maintained in Dulbecco Modified Eagle Medium (DMEM) and Human prostate adenocarcinoma (LNCaP) cells were maintained in RPMI 1640 medium. All the cells were supplemented with 10% heat-inactivated fetal bovine serum (FBS), 50 units/ml of penicillin and 50 μg/ml streptomycin. 22Rv1 cells were also supplemented with 1mM of Sodium Pyruvate. Cells were grown in humidified condition at 37°C and 5% CO_2_. Cells (5 × 10^5^) were transiently transfected with 100 nM siRNA and INTERFERin transfection reagent (Polyplus transfection, USA) according to the manufacturer's protocol for 48 h or transfected with plasmid expressing HA-tagged PIM1 by using jetPEI (Polyplus transfection, USA) for 24 h according to the manufacturer's protocol. For infection, 5 μl of concentrated lentivirus expressing PIM1 (virus titer 2 × 10^8^ pfu) was added to media containing cells at 30% confluence together with polybrene 8 μg/ml (Sigma-Aldrich, USA) for 36 h. After indicated times, cells were harvested and analyzed by western blot or by RT-qPCR. The RNAi target sequences were as follows:

PIM1 (5′-ACAUUUACAACUCAUUCCA-3′), RPS19 (5′-GGGACAAAGAGAUCUGGAC-3′), RPS6 (5′-UAUUUAAGGGCUUUCUUAC-3′), RPL7a (5′-UUGUUCUCCACCAAGGUGGUG-3′) and Control (5′-GACACGCGACUUGUACCAC-3′).

### RNA isolation and quantitative RT-PCR

For real-time RT–PCR, total RNA was extracted from transfected cells using EuroGOLD Trifast (Euroclone, Italy), according to the manufacturer's protocol. RNA was reverse transcribed into single-strand cDNA, using Moloney murine leukemia virus reverse transcriptase (Promega, Italy) and random primers (Invitrogen, Italy). cDNA was diluted at a concentration of 50 ng/ml in nuclease-free water and stored in aliquots at −80°C until used. Gene expression was detected in triplicate using SYBR Green mix (GeneSpin, Italy) on a StepOne real-Time PCR machine (Applied Biosystems, Italy), the cDNA as the template and the primer mix of interest as follows: GAPDH Fwd 5′-ACCAGGGCTGCTTTTAACTCTGGT-3′; Rev 5′-GCAAATTTCCATGGCACCGTCAAGG-3′, PIM1 Fwd 5′-TTTCGACGATGACGAAGAGA-3′; Rev 5′-GGGCCAAGCACCATCTAAT-3′, and MDM2 Fwd 5′-GAAAGAGCACAGGAAAATA-3′; Rev 5′-AAAGGAAAGGGAAATACTA-3′; RPS19 Fwd 5′-CAGCGCGGCACCTGTACCT, Rev 5′- GCTGGGCATGACGCCGTTTC

The amount of mRNA was determined using the 2^−ΔΔCt^ method considering the threshold cycle (Ct) of the sample relative to the internal reference GAPDH (ΔCt) and to untreated cells (ΔΔCt).

### Western blot analysis

Transfected or transduced cells were lysed in lysis buffer containing 350 mM NaCl, 1 mM MgCl_2_, 50 mM Tris–HCl (pH 7.5), 0.5 mM EDTA, 0.1 mM EGTA, 1% NP-40, aprotinin 1 mg/ml, phenylmethylsulfonyl fluoride 100 mg/ml and 1% [vol/vol] phosphatase inhibitor cocktail II and III from sigma). Protein concentration was measured by Bio-Rad Bradford reagent. Protein sample were prepared by addition of Laemli Sample buffer and resolved on 8-10% SDS-PAGE (Sodium dodecyl sulfate–polyacrylamide gel), transferred onto nitrocellulose Protran membrane (Schleicher and Schuell, Italy), and incubated with the following primary antibodies and antisera: mouse monoclonal antibody specific for RPS19 (Orru et al., 2007), mouse monoclonal anti-GAPDH (Millipore), mouse monoclonal anti-PIM1 (Santa Cruz Biotechnology, USA), mouse monoclonal anti-p53 (Santa Cruz Biotechnology, USA), mouse monoclonal anti-MDM2 (kindly provided by Prof. Fabiola Moretti), rabbit polyclonal anti-phospho MDM2 Ser166 (Cell Signaling), rabbit polyclonal anti-phospho AKT Ser473 (Cell Signaling Technologies, USA), rabbit polyclonal anti-Total AKT (Cell Signaling Technologies, USA), rabbit polyclonal anti-MDM4 (Bethyl laboratories), rabbit polyclonal anti-p21 (Santa Cruz Biotechnology, USA), mouse monoclonal anti-β-Actin (Santa Cruz Biotechnology, USA), rabbit polyclonal anti-RPS6 (Santa Cruz Biotechnology, USA), rabbit polyclonal anti-RPL7a (provided by Giulia Russo, Naples), rabbit polyclonal anti-Neomycin Phosphotransferase II (NPT) (Upstate) and goat polyclonal anti-Lamin (Santa Cruz Biotechnology, USA), mouse monoclonal anti-phospho BAD Ser112 (Cell Signaling Technologies, USA). Primary antibodies were revealed using horseradish peroxidase-conjugated anti-goat, anti-rabbit or anti-mouse Ab (Jackson Immunoresearch) and the ECL Clarity Western substrate detection (BIO-RAD). Quantification analyses were performed by LAS3000 Image System (Fuji) and ImageQuant software (GE Healthcare).

### Ribosome sedimentation

To separate polysomes and ribosomal subunits, HCT116 and HEK293 cells transfected with HA-PIM1, were treated with the crosslinking-inducing agent (dithiobis(succinimidyl propionate)) (DSP) at concentration of 2.5 mM and incubated for 15 min at 37°C, then the DSP was quenched by adding Tris–HCl (pH 8.0) to a final concentration of 100 mM. After 15 min incubation at room temperature, cells were collected by scraping and resuspended in 300 μl of lysis buffer containing 10 mM Tris–HCl (pH 7.5), 10 mM NaCl and 10 mM MgCl_2_, kept for 15 min on ice and then centrifuged at 16 000 g in a microcentrifuge for 15 min at 4°C. Volume of cytoplasmic extract was made up to 1 ml by adding lysis buffer and then loaded onto 30% sucrose cushion (1 ml) for centrifugation. Samples were centrifuged in a Beckman 70.1 TI rotor for 3 h at 100 000 *g*. Supernatant (S) was collected into new eppendorf as the S fraction and Pellet (P) was washed with 1 ml of 1X PBS and again centrifuged at 100 000 *g* for 45 min. After centrifugation, Pellet (P) was kept for drying and then resuspended in 30 μl of 2X loading buffer. Collected Supernatant (S) was precipitated in 200 μl of 100% trichloroacetic acid (TCA), kept for 15 min on ice and then centrifuged at 16 000 for 30 min. The precipitated pellet was washed with 5% TCA and with 1 ml of acetone and then was dried and resuspended in loading Buffer for analysis by Western blot. Both P and S fractions were loaded to a 10% SDS PAGE.

### Cell proliferation assay

HCT cells were transduced with lentivirus as described above in 24 well plates and then counted and seeded in triplicate at 10 000 cells/well in 96 multi-well plates and allowed to adhere. Cells were then treated with Doxorubicin (Sigma-Aldrich, USA) at 1 μM, Cisplatin (Sigma-Aldrich, USA) at 50 μM, Actinomycin D (Sigma-Aldrich, USA) at 50 nM or Nocodazole (Sigma-Aldrich, USA) 1 μM for 48 h. After the indicated times, cell viability was assessed by adding 20 μl of filter sterilized MTT (5 mg/ml in PBS). Following a 4 h incubation period with MTT, media was removed by syringe and the blue formazan crystals trapped in cells were dissolved in sterile DMSO (200 μl) by incubating at 37°C for 1 h. The absorbance at 570 nm was measured with a plate reader. The proliferation graph was constructed by plotting absorbance (blanked with DMSO) against time.

### Immunofluorescence staining

MCF7 cells were transfected with siRNA as described above and seeded in a 35 mm dish. After 48 h, cells were washed with PBS, fixed with 3.7% formaldehyde for 15 min at 37°C, permeabilized with 0.05% of TritonX-PBS for 5 min. Then cells were blocked with 5% bovine serum albumin (BSA) for 1 h at room temperature and washed twice with 1X PBS. After washing, cells were incubated overnight at 4°C with the primary antibodies mouse α-MDM2 monoclonal (AB-1 EMD Millipore) and rabbit α-p53 (FL-393 Santa Cruz), and then incubated with fluorescein (FITC) – conjugated AffiniPure Donkey Anti-Rabbit igG (H+L) and Rhodamine (TRITC)-conjugated AffiniPure Donkey Anti-Mouse IgG (H+L) and DAPI (Life Technologies). Cells were examined under a fluorescent microscope (Leica SP5).

### Isolation of cytosolic and nuclear fractions

Cell pellets from transfected HCT were resuspended in 400 μl of hypotonic buffer A (10 mM HEPES [pH 7.9], 10 mM KCl, 0.1 mM EDTA, 0.1 mM EGTA, 1 mM dithiothreitol, 25 mM NaF, 1 mMNaO_3_V, 0.1 mM phenylmethylsulfonyl fluoride [PMSF], leupeptin 1 mg/ml, pepstatinA 1 mg/ml, phenylmethylsulfonyl fluoride 100 mg/ml) and 1% [vol/vol] phosphatase inhibitor cocktail I) by gentle pipetting up and down and incubated in ice for 15 min. After resuspension, NP-40 was added to a final concentration of 0.6% and vortexed vigorously for 15 sec. The samples were then centrifuged at 16 000 *g* for 1 min at 4°C, and the supernatants were collected as cytosolic fractions. The pellets were washed twice with PBS and then resuspended in 60 μl of nuclei extraction buffer B (20 mM HEPES[pH 7.9], 0.4 M NaCl, 25% glycerol, 0.1 mM EDTA, 0.1 mM EGTA, 1 mM dithiothreitol, 25 mM NaF, 1 mMNaO_3_V, 0.1 mM phenylmethylsulfonyl fluoride [PMSF], leupeptin 1 mg/ml, pepstatinA 1 mg/ml, phenylmethylsulfonyl fluoride 100 mg/ml) and 1% [vol/vol] phosphatase inhibitor cocktail I) by gentle pipetting up and down. The samples were agitated for 15 min at 4°C. After agitation, samples were centrifuged at 16000 g for 5 min at 4°C and the supernatants were collected as nuclear fractions.

### Statistical analysis

Values are generally presented as the mean ± standard error of at least three independent experiments. Where indicated, data were evaluated using the Student's t test. P<0.05, P<0.01 or P<0.001 were considered to indicate statistically significant differences between values.

## SUPPLEMENTARY FIGURES


