# Supplementary material for: PIM1 destabilization activates a p53-dependent response to ribosomal stress in cancer cells
Source: Oncotarget. 2016 Mar 14;7(17):23837–49. doi: 10.18632/oncotarget.8070 (PMC5029667; doi:10.18632/oncotarget.8070)
Supplement: Supplementary file 1 [file oncotarget-07-23837-s001.pdf]

PIM1 destabilization activates a p53-dependent response to ribosomal stress in cancer cells

SUPPLEMENTARY FIGURES

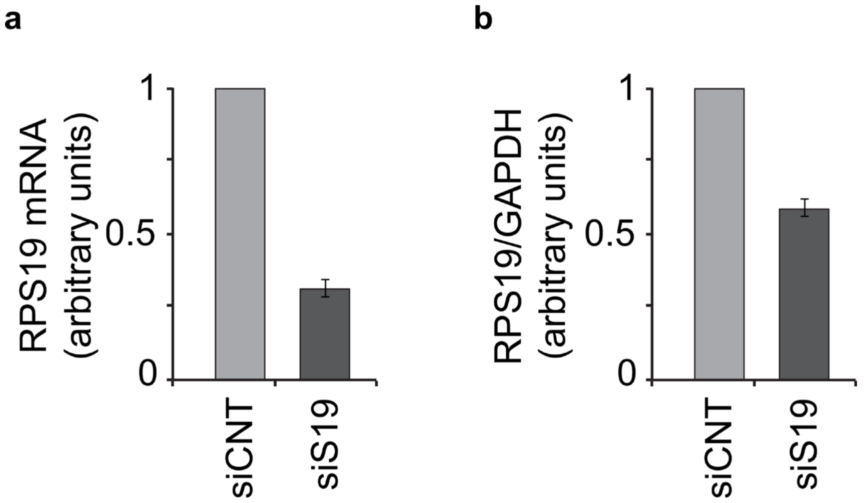

Supplementary Figure S1: RPS19 mRNA and protein levels. a. Total RNA was extracted from HCT116 cells transfected with control siRNA (siCNT) or with RPS19-specific siRNA (siS19) and analyzed by qRT-PCR with primers specific for RPS19 and GAPDH. The results of RT–qPCR from three independent RNA preparations, each one analyzed in triplicate, are reported as a column plot of the mean±s.e.m. of RPS19 mRNA relative to GAPDH mRNA. **b.** Total extracts were analyzed by western blot with primary antibodies specific for RPS19 and GAPDH. Quantification of proteins from seven independent experiments are reported as a column plot of the mean±s.e.m. of the densitometry values normalized first by GAPDH and then by siCNT value.

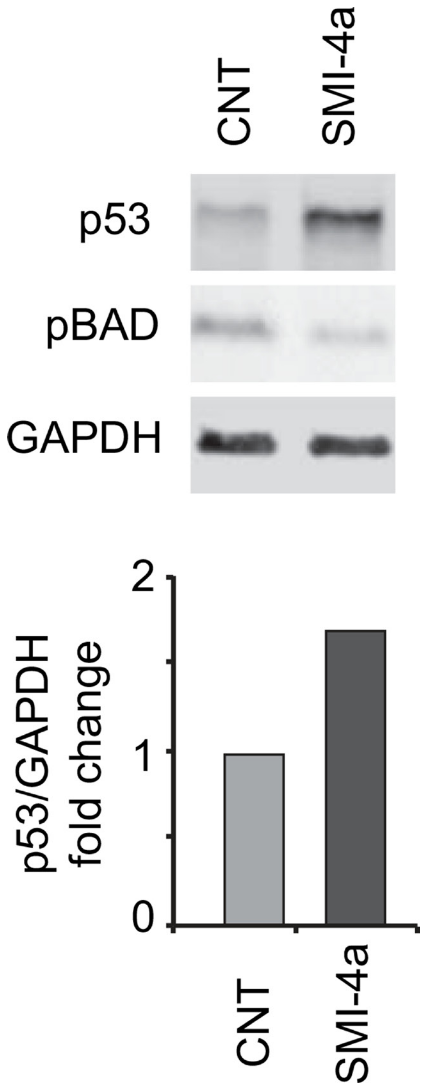

Supplementary Figure S2: Effect of PIM inhibitor SMI-4a on p53 level. HCT116 cells were treated with PIM inhibitor SMI-4a at 50µM for 4hr in absence of serum. Total protein extracts were analyzed by western blot with indicated primary antibodies. Column plot represents the average of two blots from the same experiment.

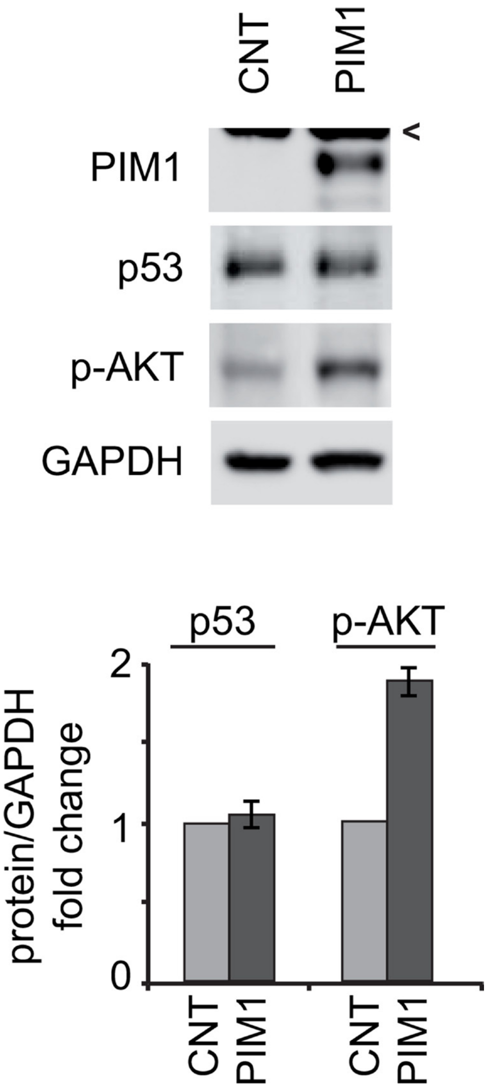

Supplementary Figure S3: PIM1 overexpression in control HCT cells. HCT116 cells were transduced with PIM1-expressing lentivirus. Total protein extracts were analyzed by western blot with the indicated primary antibodies. Quantification of proteins from at least three independent experiments are reported as a column plot of the mean±s.e.m. of the values normalized by GAPDH. <, non-specific band.

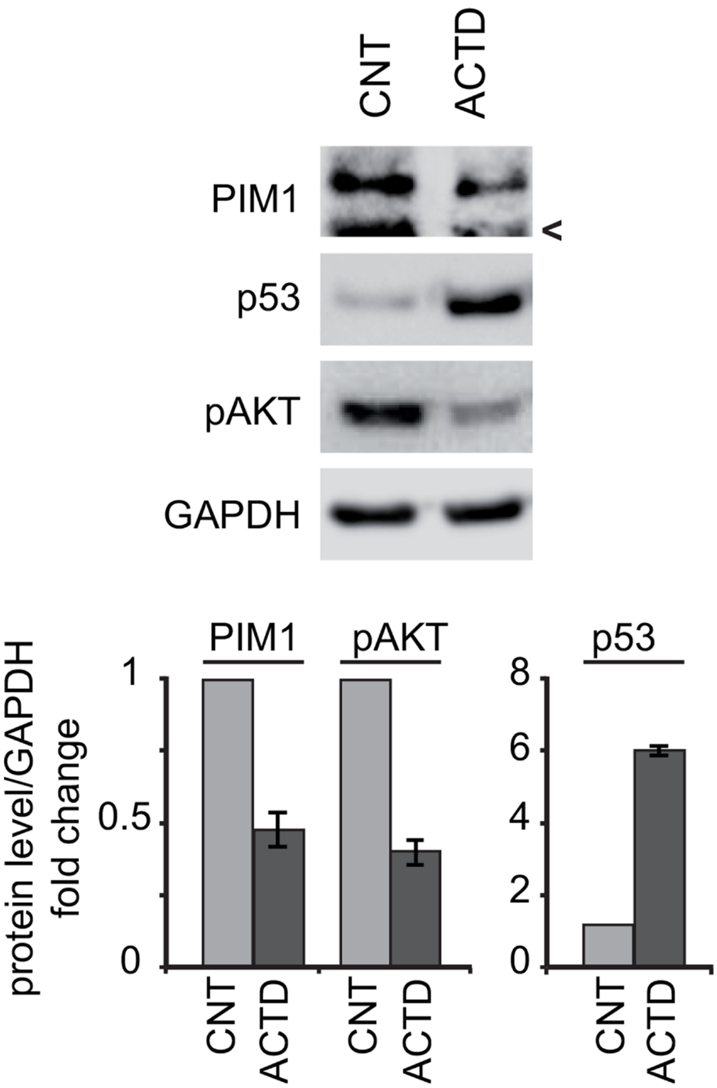

Supplementary Figure S4: Protein levels after Actinomycin D treatment. HCT116 cells were treated for 4 hrs with 50 nM Actinomycin D. Total protein extracts were analyzed by western blot with indicated primary antibodies. Column plots report the mean of three experiments ±s.e.m.
